# Design of Adaptive LQR Control Based on Improved Grey Wolf Optimization for Prosthetic Hand

**DOI:** 10.3390/biomimetics10070423

**Published:** 2025-06-30

**Authors:** Khaled Ahmed, Ayman A. Aly, Mohamed O. Elhabib

**Affiliations:** 1 Department of Mechanical Engineering, Faculty of Engineering, King Abdulaziz University, Jeddah 21589, Saudi Arabia; 2 Department of Mechanical Engineering, College of Engineering, Taif University, Taif 21944, Saudi Arabia; 3 Control and Mechatronics Engineering Division, Faculty of Electrical Engineering, Universiti Teknologi Malaysia, Johor Bahru 81310, Malaysia; osman@graduate.utm.my; 4 King Salman Center for Disability Research, Riyadh 11614, Saudi Arabia

**Keywords:** prosthetic hand, disabled people, IGWO, optimal control

## Abstract

Assistive technologies, particularly multi-fingered robotic hands (MFRHs), are critical for enhancing the quality of life for individuals with upper-limb disabilities. However, achieving precise and stable control of such systems remains a significant challenge. This study proposes an Improved Grey Wolf Optimization (IGWO)-tuned Linear Quadratic Regulator (LQR) to enhance the control performance of an MFRH. The MFRH was modeled using Denavit–Hartenberg kinematics and Euler–Lagrange dynamics, with micro-DC motors selected based on computed torque requirements. The LQR controller, optimized via IGWO to systematically determine weighting matrices, was benchmarked against PID and PID-PSO controllers under diverse input scenarios. For step input, the IGWO-LQR achieved a settling time of 0.018 s with zero overshoot for Joint 1, outperforming PID (settling time: 0.0721 s; overshoot: 6.58%) and PID-PSO (settling time: 0.042 s; overshoot: 2.1%). Similar improvements were observed across all joints, with Joint 3 recording an IAE of 0.001334 for IGWO-LQR versus 0.004695 for PID. Evaluations under square-wave, sine, and sigmoid inputs further validated the controller’s robustness, with IGWO-LQR consistently delivering minimal tracking errors and rapid stabilization. These results demonstrate that the IGWO-LQR framework significantly enhances precision and dynamic response.

## 1. Introduction

According to the World Health Organization, more than 2.5 billion people need one or more assistive products. Assistive technology (AT) refers to products, equipment, and systems that enhance learning, working, and daily living for people with disabilities [1]. Losing a human arm impacts a person’s life by reducing their ability to perform daily activities.

Biomechatronics represents a sophisticated, interdisciplinary field that integrates principles from neuroscience, robotics, mechanics, and biology [2]. A primary objective within this domain is the development of advanced devices engineered to augment and replicate natural human motion. These innovations are instrumental in facilitating and expediting the recovery of motor control in patients with physical impairments [2,3]. A biomechatronic system is conceptually analogous to its human counterpart, requiring components that emulate the functions of muscles, neurons, and the brain. In this framework, electric motors serve as actuators, analogous to biological muscles [4]; an array of sensors functions as the neural network, gathering sensory data [5]; and a central microcontroller operates as the processing unit. This controller mirrors the cognitive functions of the brain by implementing control strategies that translate sensor data into commands for the actuators [6]. This paradigm is exemplified in the design of robotic appendages, such as hands, where the mechanics of movement are meticulously modeled to replicate the dexterity and functionality of a human hand [7]. Consequently, biomechatronics stands at the vanguard of contemporary robotics. A key component in these systems is the “gripper”, which is the terminal effector responsible for establishing physical contact and manipulating objects [8]. It ensures place and direction when the object is held and attached to a handling device.

An anthropomorphic robot hand is an advanced end-effector designed to replicate the complex manipulative capabilities of the human hand [9]. In particular, multi-fingered robotic hands (MFRHs) are of growing significance in fields such as advanced manufacturing, assistive robotics, and other applications where stable grasping and dexterous manipulation are critical [10,11]. The development of high-fidelity models of the human hand and fingers has become a focal point of extensive research. This attention is driven by the substantial potential for practical applications in diverse domains, including medical rehabilitation, advanced prosthetics, human–computer interaction (HCI), and human–machine interface design [12,13]. The efficacy of these biomimetic models is contingent upon the accurate representation of the hand’s anatomical structure, functional capabilities, and specific kinematic and dynamic properties [14]. Furthermore, the intended application fundamentally governs the design choices and complexity of the model [15].

The kinematics of the human arm is characterized by high dimensionality, possessing a large number of degrees of freedom (DOFs). To achieve computational tractability and simplify control, human arm motion is frequently represented in robotic systems with a reduced number of DOFs, a modeling approach that seeks to capture the most significant aspects of natural movement while reducing complexity [16,17]. Prosthetic-arm rejection depends on the level of amputation; the closer the amputation, the higher the rejection rate [18]. The key motivators for the abandonment of prosthetics include technical restrictions of the system, discomfort, and appearance [19]. Currently, there are several commercial solutions for people with upper-limb amputations, but they are not practical for real life, and other solutions are not affordable for most of the population.

The core focus of many researchers in the past three decades has been grasping, and it has been studied from multiple perspectives. Important topics in this context include modeling human fingers and control [17,18]; grip-quality assessment [19,20]; gripping in multi-task systems such as collaborative robotic manipulators [21]; robot grippers [22]; and specific features of gripping, such as stable grip [23], shape-closure grip [24], finger-movement coordination [17,18], virtual hand modeling and simulation [19,20,21,22], articulated human hands [23,24], and force-closure grip [25,26]. In most cases, this type of robot hand has four/five fingers or fewer [27,28].

This work confronts the fundamental challenge of achieving high-precision control in MFRHs by introducing a novel optimal-control framework. The primary objective is to design and validate an adaptive Linear Quadratic Regulator (LQR) whose performance is systematically enhanced by an Improved Grey Wolf Optimization (IGWO) algorithm. The core concern of this proposed control system is to eliminate position errors, minimize overshoot, and suppress disturbances to ensure the robotic hand follows a desired trajectory with high fidelity.

To achieve this, the study pursues the following specific aims:

- First, a comprehensive, control-oriented mathematical model of the prosthetic finger is proposed. This model utilizes Denavit–Hartenberg (D-H) parameters for kinematic analysis and the Euler–Lagrange formulation for dynamics, providing the essential foundation for controller design and simulation.
- Second, the IGWO algorithm is employed to systematically optimize the LQR controller’s Q and R weighting matrices. This approach addresses the critical challenge of manual tuning, ensuring an optimal trade-off between tracking precision and energy consumption, which is reflected in the controller’s ability to minimize the Integral Absolute Error (IAE).
- Finally, the proposed IGWO-LQR controller is comprehensively benchmarked against both a conventional PID controller and a PID-PSO (Particle Swarm Optimization) controller to validate its superiority in performance, speed, and robustness across diverse input signals.

This paper is organized as follows. Section 2 presents the mathematical model of the finger, detailing the three-degree-of-freedom (DOF) kinematic and dynamic formulations. Section 3 describes the design of the LQR controller and the IGWO algorithm used for its optimization. The comparative results of the controllers under various input scenarios are presented and analyzed in Section 4. Finally, Section 5 provides the conclusions of this study.

## 2. Multi-Fingered Robot Hand (MFRH)

**Figure 1:**
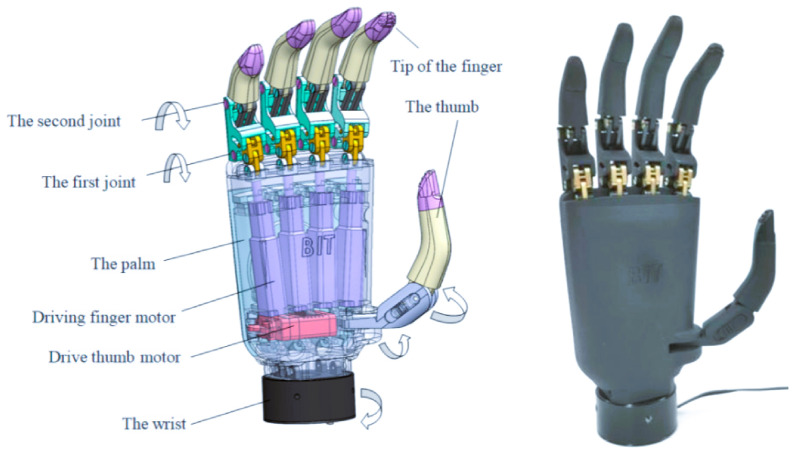
Multi-fingered robotic hand (MFRH).

A mathematical model is essential for designing, simulating, and controlling a multi-fingered robot hand. In this work, the MFRH as in Figure 1 is modeled by integrating (i) kinematic analysis, (ii) dynamic formulation, and (iii) actuator selection and modeling. The approach is based on the Denavit–Hartenberg (DH) convention for kinematics and the Euler–Lagrange formulation for dynamics. The required joint torques, which are computed from the dynamic equations, serve as the basis for selecting appropriate micro-DC motors and planetary gearheads [29].

### 2.1. Kinematic Model

Each finger of the MFRH is modeled as a serial chain with three active revolute joints corresponding to the metacarpophalangeal joints (MCP), the proximal interphalangeal joints (PIP), and the distal interphalangeal joints (DIP). The DH parameters for a typical finger are summarized in Table 1. For each joint *i*, the angle of the joint is indicated by $\theta_{i}$; the offset $d_{i}$ is set to zero; the length of the link $a_{i}$ is given (with $a_{2}=l_{1}$, $a_{3}=l_{2}$, and $a_{4}=l_{3}$); and the twist angle $\alpha_{i}$ is zero [29].

#### 2.1.1. Forward Kinematics

The homogeneous transformation matrices for adjacent frames are derived using DH parameters. For joint *i*, the transformation matrix $H_{i-1}^{i}$ is

(1) $$\begin{array}{c} \begin{array}{c} H_{i-1}^{i}=\left[\begin{array}{cccc} \cos\theta_{i} & -\sin\theta_{i} & 0 & a_{i-1}\\ \sin\theta_{i}\cos\alpha_{i-1} & \cos\theta_{i}\cos\alpha_{i-1} & \sin\alpha_{i-1} & d_{i}\sin\alpha_{i-1}\\ \sin\theta_{i}\sin\alpha_{i-1} & \cos\theta_{i}\sin\alpha_{i-1} & \cos\alpha_{i-1} & d_{i}\cos\alpha_{i-1}\\ 0 & 0 & 0 & 1 \end{array}\right] \end{array} \end{array}$$

For the MFRH finger, the transformations are as follows:

For Joint 1:

(2) $$\begin{array}{c} \begin{array}{c} H_{0}^{1}=\left[\begin{array}{cccc} \cos\theta_{1} & -\sin\theta_{1} & 0 & 0\\ \sin\theta_{1} & \cos\theta_{1} & 0 & 0\\ 0 & 0 & 1 & 0\\ 0 & 0 & 0 & 1 \end{array}\right] \end{array} \end{array}$$

For Joint 2:

(3) $$\begin{array}{c} \begin{array}{c} H_{1}^{2}=\left[\begin{array}{cccc} \cos\theta_{2} & -\sin\theta_{2} & 0 & l_{1}\\ \sin\theta_{2} & \cos\theta_{2} & 0 & 0\\ 0 & 0 & 1 & 0\\ 0 & 0 & 0 & 1 \end{array}\right] \end{array} \end{array}$$

For Joint 3:

(4) $$\begin{array}{c} \begin{array}{c} H_{2}^{3}=\left[\begin{array}{cccc} \cos\theta_{3} & -\sin\theta_{3} & 0 & l_{2}\\ \sin\theta_{3} & \cos\theta_{3} & 0 & 0\\ 0 & 0 & 1 & 0\\ 0 & 0 & 0 & 1 \end{array}\right] \end{array} \end{array}$$

End-effector (DIP):

(5) $$\begin{array}{c} \begin{array}{c} H_{3}^{4}=\left[\begin{array}{cccc} 1 & 0 & 0 & l_{3}\\ 0 & 1 & 0 & 0\\ 0 & 0 & 1 & 0\\ 0 & 0 & 0 & 1 \end{array}\right] \end{array} \end{array}$$

The position of the end-effector relative to the base frame is:

(6) $$\begin{array}{c} \begin{array}{c} H_{0}^{4}=H_{0}^{1}\cdot H_{1}^{2}\cdot H_{2}^{3}\cdot H_{3}^{4} \end{array} \end{array}$$

After simplification, the position coordinates of the fingertip are:

(7) $$\begin{array}{c} \begin{array}{c} x=l_{1}\cos\theta_{1}+l_{2}\cos\left(\theta_{1}+\theta_{2}\right)+l_{3}\cos\left(\theta_{1}+\theta_{2}+\theta_{3}\right) \end{array} \end{array}$$

(8) $$\begin{array}{c} \begin{array}{c} y=l_{1}\sin\theta_{1}+l_{2}\sin\left(\theta_{1}+\theta_{2}\right)+l_{3}\sin\left(\theta_{1}+\theta_{2}+\theta_{3}\right) \end{array} \end{array}$$

#### 2.1.2. Inverse Kinematics

For a desired end-effector position (x,y),

(9) $$\begin{array}{c} \begin{array}{c} \theta_{2}=\arctan_{2}\left(\pm \sqrt{1-\cos^{2}\theta_{2}},\cos\theta_{2}\right) \end{array} \end{array}$$

(10) $$\begin{array}{c} \begin{array}{c} \theta_{1}=\arctan_{2}\left(y,x\right)-\arctan_{2}\left(k_{2},k_{1}\right) \end{array} \end{array}$$

(11) $$\begin{array}{c} \begin{array}{c} \theta_{3}=\phi -\theta_{1}-\theta_{2}=\arctan_{2}\left(S_{\phi },C_{\phi }\right)-\theta_{1}-\theta_{2} \end{array} \end{array}$$

where $S_{\phi }=\sin\phi$ and $C_{\phi }=\cos\phi$.

### 2.2. Dynamic Model

**Figure 2:**
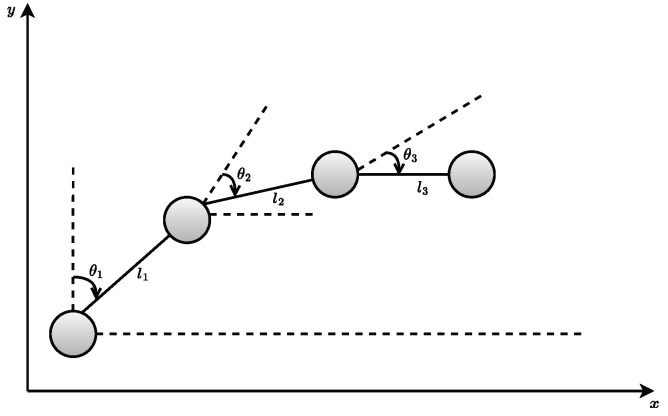
Angles of flexion of one finger.

The dynamic model derives the equations of motion using the Euler–Lagrange formulation, based on the finger-linkage model illustrated in Figure 2.

#### 2.2.1. Kinetic Energy (*K*)

The total kinetic energy includes translational and rotational components for each link. For link *i*, the angular velocity is denoted by $\omega_{1}={\dot{\theta }}_{1}$, $\omega_{2}={\dot{\theta }}_{1}+{\dot{\theta }}_{2}$, $\omega_{3}={\dot{\theta }}_{1}+{\dot{\theta }}_{2}+{\dot{\theta }}_{3}$. The total kinetic energy in matrix form is:

(12) $$\begin{array}{c} \begin{array}{c} K=\frac{1}{2}{\dot{\theta }}^{T}A\left(\theta \right)\dot{\theta } \end{array} \end{array}$$

where A(θ) is the symmetric inertia matrix with the following elements:

(13) $$\begin{array}{c} \begin{array}{c} \begin{array}{c} A_{11}=\frac{1}{4}m_{1}l_{1}^{2}+m_{2}\left(l_{1}^{2}+\frac{1}{4}l_{2}^{2}+l_{1}l_{2}\cos\theta_{2}\right)+\\ m_{3}\left(l_{1}^{2}+l_{2}^{2}+\frac{1}{4}l_{3}^{2}+2l_{1}l_{2}\cos\theta_{2}+l_{1}l_{3}\cos\left(\theta_{2}+\theta_{3}\right)+l_{2}l_{3}\cos\theta_{3}\right) \end{array} \end{array} \end{array}$$

(14) $$\begin{array}{c} \begin{array}{c} \begin{array}{c} A_{12}=A_{21}=\frac{1}{2}m_{2}\left(\frac{1}{2}l_{2}^{2}+l_{1}l_{2}\cos\theta_{2}\right)+\\ m_{3}\left(l_{2}^{2}+\frac{1}{4}l_{3}^{2}+l_{1}l_{2}\cos\theta_{2}+\frac{1}{2}l_{1}l_{3}\cos\left(\theta_{2}+\theta_{3}\right)+l_{2}l_{3}\cos\theta_{3}\right) \end{array} \end{array} \end{array}$$

(15) $$\begin{array}{c} \begin{array}{c} A_{13}=A_{31}=\frac{1}{2}m_{3}\left(\frac{1}{2}l_{3}^{2}+l_{1}l_{3}\cos\left(\theta_{2}+\theta_{3}\right)+l_{2}l_{3}\cos\theta_{3}\right) \end{array} \end{array}$$

(16) $$\begin{array}{c} \begin{array}{c} A_{22}=\frac{1}{4}m_{2}l_{2}^{2}+m_{3}\left(l_{2}^{2}+\frac{1}{4}l_{3}^{2}+l_{2}l_{3}\cos\theta_{3}\right) \end{array} \end{array}$$

(17) $$\begin{array}{c} \begin{array}{c} A_{23}=A_{32}=\frac{1}{2}m_{3}\left(\frac{1}{2}l_{3}^{2}+l_{2}l_{3}\cos\theta_{3}\right) \end{array} \end{array}$$

(18) $$\begin{array}{c} \begin{array}{c} A_{33}=\frac{1}{4}m_{3}l_{3}^{2} \end{array} \end{array}$$

#### 2.2.2. Potential Energy (*P*)

The gravitational potential energy for each link is as follows:

(19) $$\begin{array}{c} \begin{array}{c} P=\sum_{i=1}^{3}m_{i}gy_{i} \end{array} \end{array}$$

(20) $$\begin{array}{c} \begin{array}{c} \begin{array}{c} P=\frac{1}{2}m_{1}gl_{1}\sin\theta_{1}+m_{2}g\left(l_{1}\sin\theta_{1}+\frac{l_{2}}{2}\sin\left(\theta_{1}+\theta_{2}\right)\right)+\\ m_{3}g\left(l_{1}\sin\theta_{1}+l_{2}\sin\left(\theta_{1}+\theta_{2}\right)+\frac{l_{3}}{2}\sin\left(\theta_{1}+\theta_{2}+\theta_{3}\right)\right) \end{array} \end{array} \end{array}$$

Equations of Motion:

Using the Lagrangian L=K−P, the torque $\tau_{i}$ for joint *i* is derived as

(21) $$\begin{array}{c} \begin{array}{c} \tau_{i}=\frac{d}{dt}\left(\frac{\partial L}{\partial {\dot{\theta }}_{i}}\right)-\frac{\partial L}{\partial \theta_{i}}\;\left(i=1,2,3\right) \end{array} \end{array}$$

Expanding for $\tau_{1}$, the following is obtained:

(22) $$\begin{array}{c} \begin{array}{c} \tau_{1}=A_{11}{\ddot{\theta }}_{1}+A_{12}{\ddot{\theta }}_{2}+A_{13}{\ddot{\theta }}_{3}+\frac{\partial A_{11}}{\partial \theta_{1}}{\dot{\theta }}_{1}^{2}+\ldots -\frac{\partial P}{\partial \theta_{1}} \end{array} \end{array}$$

Similar expansions apply for $\tau_{2}$ and $\tau_{3}$. The required torques for the finger joints are calculated from these dynamic equations, and are approximately 0.8307 Nm for Joint 1, 0.6955 Nm for Joint 2, and 0.1589 Nm for Joint 3.

### 2.3. Actuator Selection and Modeling

To achieve the required torques, appropriate micro-DC motors and gearheads were selected based on the computed dynamic requirements.

#### Torque and Speed Derivation

For Joint 1, which requires a torque of 0.8307 Nm, a Maxon EC-max 22 Brushless DC Motor was chosen. This motor operates at a nominal voltage of 24 V, offers a no-load speed of 10,500 rpm, and provides a maximum continuous torque of 24 mNm. When paired with a Maxon GP 22 C Planetary Gearhead with a reduction ratio of 64:1 and an efficiency of approximately 70%, the combined system produces an output torque of roughly 1.075 Nm and reduces the output speed to about 164 rpm.

For Joint 2, which requires 0.6955 Nm, the same motor is used, but the gearhead reduction ratio is adjusted to 56:1. This configuration results in an output torque of approximately 0.94 Nm and an output speed of around 188 rpm.

For Joint 3, with a lower torque requirement of 0.1589 Nm, a smaller Maxon EC 16 Brushless DC Motor was selected. This motor has a no-load speed of 15,500 rpm and a maximum continuous torque of 9 mNm. Coupling it with a Maxon GP 16 A Planetary Gearhead with a reduction ratio of 43:1 (and an efficiency of approximately 70%) yields an output torque of about 0.271 Nm and an output speed of approximately 360 rpm. The output torque $T_{\mathrm{out}}$ and speed $\omega_{\mathrm{out}}$ of the motor–gearhead system are calculated as follows:

(23) $$\begin{array}{c} \begin{array}{cc} T_{\mathrm{out}} & =T_{\mathrm{motor}}\cdot N\cdot \eta \end{array} \end{array}$$

(24) $$\begin{array}{cc} \omega_{\mathrm{out}} & =\frac{\omega_{\mathrm{motor}}}{N} \end{array}$$

where *N* is the gear ratio, η is the gearhead efficiency, $T_{\mathrm{motor}}$ is the motor’s continuous torque, and $\omega_{\mathrm{motor}}$ is the motor’s no-load speed.

Joint 1:

For Joint 1, the calculated torque is $T_{1\mathrm{out}}=1.075\;\mathrm{Nm}$, and $\omega_{1\mathrm{out}}=164.06\;\mathrm{rpm}$. A Maxon EC-max 22 motor with a GP 22 C gearhead (64:1 ratio) satisfies this requirement.

Joint 2:

For Joint 2, the calculated torque is $T_{2\mathrm{out}}=0.94\;\mathrm{Nm}$, and $\omega_{2\mathrm{out}}=187.5\;\mathrm{rpm}$. The same EC-max 22 motor is used with a GP 22 C gearhead adjusted to 56:1.

Joint 3:

For Joint 3, the calculated torque is $T_{3\mathrm{out}}=0.271\;\mathrm{Nm}$, and $\omega_{3\mathrm{out}}=60.47\;\mathrm{rpm}$. A Maxon EC 16 motor with a GP 16 A gearhead (43:1 ratio) was selected, providing a 1.7× safety margin relative to the required torque.

The actuator dynamics are governed by the standard motor equations. The voltage equation is given by

(25) $$\begin{array}{c} \begin{array}{c} V=R\cdot I+L\cdot \frac{dI}{dt}+K_{e}\cdot \omega \end{array} \end{array}$$

and the torque generated by the motor is

(26) $$\begin{array}{c} \begin{array}{c} T_{m}=K_{t}\cdot I \end{array} \end{array}$$

Furthermore, the mechanical dynamics are represented as

(27) $$\begin{array}{c} \begin{array}{c} T_{m}-T_{\mathrm{load}}=J_{\mathrm{total}}\cdot \frac{d\omega }{dt}+B\cdot \omega +T_{f} \end{array} \end{array}$$

The dynamic behavior of each motor-driven joint, as described by the electromechanical equations, can be represented using a state-space model, which is essential for modern control-design techniques like the Linear Quadratic Regulator (LQR). To control the angular position of the joint, we define a state vector x(t), an input u(t), and an output y(t).

The state variables are selected to represent the key dynamic properties of the motor:

$x_{1}=\theta \left(t\right)$: the angular position of the joint.
$x_{2}=\omega \left(t\right)=\dot{\theta }\left(t\right)$: the angular velocity of the joint.
$x_{3}=I\left(t\right)$: the armature current of the motor.
The control input u(t) is the voltage applied to the motor, V(t), and the system output y(t) is the angular position, θ(t).

From the governing equations for voltage and mechanical dynamics, we can derive the following first-order differential equations, assuming that the load torque $T_{load}$ and static friction $T_{f}$ are treated as external disturbances:

1. ${\dot{x}}_{1}=\dot{\theta }=\omega =x_{2}$
2. ${\dot{x}}_{2}=\dot{\omega }=\frac{1}{J_{total}}\left(T_{m}-B\omega \right)=\frac{K_{t}}{J_{total}}I-\frac{B}{J_{total}}\omega =\frac{K_{t}}{J_{total}}x_{3}-\frac{B}{J_{total}}x_{2}$
3. ${\dot{x}}_{3}=\dot{I}=\frac{1}{L}\left(V-RI-K_{e}\omega \right)=\frac{1}{L}V-\frac{R}{L}I-\frac{K_{e}}{L}\omega =\frac{1}{L}u-\frac{R}{L}x_{3}-\frac{K_{e}}{L}x_{2}$

These equations can be written in the standard state-space form $\dot{x}\left(t\right)=Ax\left(t\right)+Bu\left(t\right)$ and y(t)=Cx(t)+Du(t):

(28) $$\begin{array}{c} \begin{array}{c} \left[\begin{array}{ccc} 0 & 1 & 0\\ 0 & -\frac{B}{J_{total}} & \frac{K_{t}}{J_{total}}\\ 0 & -\frac{K_{e}}{L} & -\frac{R}{L} \end{array}\right]\left[\begin{array}{c} \theta \\ \omega \\ I \end{array}\right]+\left[\begin{array}{c} 0\\ 0\\ \frac{1}{L} \end{array}\right]V\left(t\right) \end{array} \end{array}$$

(29) $$\begin{array}{c} \begin{array}{c} y\left(t\right)=\left[\begin{array}{ccc} 1 & 0 & 0 \end{array}\right]\left[\begin{array}{c} \theta \\ \omega \\ I \end{array}\right] \end{array} \end{array}$$

This state-space representation forms the mathematical basis for the LQR controller design discussed in the following section, where $J_{\mathrm{total}}$ includes contributions from the motor, gearhead, and load inertia; *B* is the viscous friction coefficient; and $T_{f}$ represents static friction. These equations are employed to simulate the motor and joint dynamics accurately. Table A1 and Table A2 in Appendix A summarize the key parameters of the motors and gearheads.

## 3. Controller Design

### 3.1. Controller Design: Linear Quadratic Regulator (LQR)

In the MFRH designed in this study, each joint of the robotic finger is actuated by a dedicated brushless DC motor. Since these actuators share identical or near-identical electromechanical characteristics, a single set of LQR controller gains was designed and subsequently applied to all three motors.

Each motor is represented by a continuous-time state-space model capturing both the electrical and mechanical subsystems. Let x(t) denote the state vector, u(t) the control input (motor voltage), and y(t) the system output (e.g., angular position). The general form is

(30) $$\begin{array}{c} \begin{array}{c} \dot{x}\left(t\right)\;=\;A\;x\left(t\right)\;+\;B\;u\left(t\right),\;y\left(t\right)\;=\;C\;x\left(t\right), \end{array} \end{array}$$

where A, B, and C are matrices derived from the motor’s electromechanical Equations (28) and (29). Key parameters—such as rotor inertia, back-EMF constant, torque constant, and winding resistance—are included to capture the dominant dynamics. Minor effects (e.g., gearhead friction, inductance) are either integrated into the model or treated as disturbances, depending on their relative magnitude.

Given the similarity in physical construction and parameter values across the three actuators, we adopt a uniform state-space representation (A,B,C). This common model underpins the decision to apply a single set of LQR gains to all three motors.

#### 3.1.1. LQR Control Formulation

**Figure 3:**
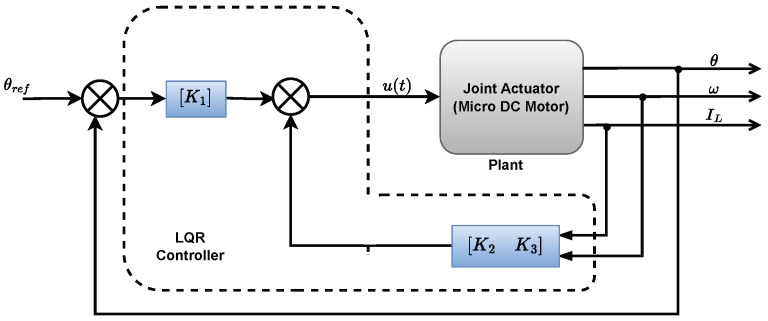
LQR schematic diagram.

To regulate the motor’s angular position and velocity while minimizing control effort, we employed a Linear Quadratic Regulator (LQR). The LQR design aims to find an optimal feedback law of the form

(31) $$\begin{array}{c} \begin{array}{c} u(t)=-K\;x(t), \end{array} \end{array}$$

where K is a constant-gain matrix. The schematic for this control structure is shown in Figure 3, where the controller gains are obtained by minimizing the following quadratic cost function:

(32) $$\begin{array}{c} \begin{array}{c} J=\int_{0}^{\infty }\left[x^{T}\left(t\right)\;Q\;x\left(t\right)+u^{T}\left(t\right)\;R\;u\left(t\right)\right]\;dt \end{array} \end{array}$$

subject to the state-space dynamics. Here, Q is the state-weighting matrix, penalizing large deviations in states (angular position, angular velocity, armature current), and R is the input-weighting matrix, penalizing excessive control effort (motor voltage).

For the cost function to be well-posed and for a stable solution to exist, these matrices must have specific properties. The state-weighting matrix Q must be a symmetric, positive, semi-definite matrix ($Q=Q^{T}$, Q≥0), ensuring that any state deviation results in a non-negative penalty. The input-weighting matrix R must be a symmetric, positive, definite matrix ($R=R^{T}$, R>0) which enforces a strict penalty on any control action and guarantees the solvability of the control problem. Typically, for a system with the state vector $x={\left[\theta ,\omega ,I\right]}^{T}$, Q is chosen as a diagonal matrix, $Q=\mathrm{diag}(q_{1},q_{2},q_{3})$, where $q_{1},q_{2},q_{3}\geq 0$ correspond to penalties for errors in angular position, angular velocity, and armature current, respectively. Since the motor has a single voltage input, R is a positive scalar. The appropriate selection of Q and R reflects the desired trade-off between precise tracking (high state penalty) and efficient energy usage (high control penalty).

#### 3.1.2. LQR Gain Calculation

The gain matrix K is obtained via the algebraic Riccati equation (ARE). Specifically, a solution P to the ARE,

(33) $$\begin{array}{c} \begin{array}{c} A^{T}P+P\;A-P\;B\;R^{-1}B^{T}\;P+Q=0 \end{array} \end{array}$$

is used to find the optimal gain. However, for the resulting control law to be optimal and to guarantee a stable system, the solution P must be the unique, symmetric, positive-definite solutionto the ARE. The existence of such a solution is ensured under standard LQR assumptions (i.e., the system is stabilizable and detectable).

This specific choice of P is critical because it guarantees that the closed-loop system matrix, $A_{cl}=\left(A-BK\right)$, is Hurwitz, meaning all of its eigenvalues have negative real parts. This ensures the asymptotic stability of the closed-loop system. This stabilizing solution yields the optimal control law:

(34) $$\begin{array}{c} \begin{array}{c} K=R^{-1}\;B^{T}\;P \end{array} \end{array}$$

Standard numerical routines (e.g., MATLAB’s R2024b lqr function) are specifically designed to efficiently compute this unique positive-definite solution P and the corresponding optimal-gain matrix K. This procedure is performed once, resulting in a single gain matrix K that is subsequently applied to each motor in the system.

### 3.2. Controller Optimization: Improved Grey Wolf Optimization

The performance of the LQR controller is highly sensitive to the choice of the weighting matrices Q and R. To systematically determine their optimal values, the Improved Grey Wolf Optimization (IGWO) algorithm was employed. IGWO is an enhanced metaheuristic algorithm that builds upon the original Grey Wolf Optimizer (GWO) by incorporating a dimension learning-based hunting (DLH) strategy. This strategy improves population diversity and mitigates premature convergence, thereby enhancing both the exploration and exploitation capabilities of the algorithm [30].

#### 3.2.1. IGWO Algorithm Formulation

The IGWO algorithm initializes a population of candidate solutions (wolves), where each wolf’s position vector X represents a potential solution within a predefined search space. The position of the *i*-th wolf in a *D*-dimensional search space is given by

(35) $$\begin{array}{c} \begin{array}{c} X_{ij}=l_{j}+\mathrm{rand}\left[0,1\right]\cdot \left(u_{j}-l_{j}\right),\;i=1,2,\ldots ,N,\;j=1,2,\ldots ,D, \end{array} \end{array}$$

where $l_{j}$ and $u_{j}$ are the lower and upper bounds for the *j*-th variable, *N* is the population size, and rand[0,1] is a uniformly distributed random number.

#### Canonical GWO Search Strategy

In GWO, the hunt is guided by the three best wolves found so far: $X_{\alpha }$ (best), $X_{\beta }$ (second-best), and $X_{\delta }$ (third-best). The encircling behavior is modeled as follows:

(36) $$\begin{array}{c} \begin{array}{cc} D & =|C⊙X_{p}\left(t\right)-X\left(t\right)|, \end{array} \end{array}$$

(37) $$\begin{array}{cc} X(t+1) & =X_{p}\left(t\right)-A⊙D. \end{array}$$

Here, X is the position vector of a wolf and $X_{p}$ is the position of the prey. The operations are element-wise: ⊙ denotes element-wise multiplication, and |·| is the element-wise absolute value. The terms A and C are *D*-dimensional coefficient vectors computed as follows:

(38) $$\begin{array}{c} \begin{array}{cc} A & =2a\cdot r_{1}-a, \end{array} \end{array}$$

(39) $$\begin{array}{cc} C & =2\cdot r_{2}, \end{array}$$

where $r_{1}$ and $r_{2}$ are random vectors with each element in [0,1]. The parameter *a* is linearly decreased from 2 to 0 over the course of iterations (*t*) to balance exploration and exploitation:

(40) $$\begin{array}{c} \begin{array}{c} a\left(t\right)=2-\frac{2t}{\mathrm{MaxIter}}. \end{array} \end{array}$$

The position of each wolf is updated based on the locations of the three leader wolves. This is achieved by calculating three potential new positions and averaging them. First, the distances to the leaders are calculated:

(41) $$\begin{array}{c} \begin{array}{cc} D_{\alpha } & =|C_{1}⊙X_{\alpha }-X\left(t\right)|,\\ D_{\beta } & =|C_{2}⊙X_{\beta }-X\left(t\right)|,\\ D_{\delta } & =|C_{3}⊙X_{\delta }-X\left(t\right)|, \end{array} \end{array}$$

where $A_{1},A_{2},A_{3}$ and $C_{1},C_{2},C_{3}$ are coefficient vectors calculated independently for each leader wolf using (38) and (39). Then, the three potential next positions are determined:

(42) $$\begin{array}{c} \begin{array}{cc} X_{1}\left(t+1\right) & =X_{\alpha }-A_{1}⊙D_{\alpha },\\ X_{2}\left(t+1\right) & =X_{\beta }-A_{2}⊙D_{\beta },\\ X_{3}\left(t+1\right) & =X_{\delta }-A_{3}⊙D_{\delta }. \end{array} \end{array}$$

The candidate position generated by the canonical GWO strategy, denoted as $X_{i}^{\mathrm{GWO}}\left(t+1\right)$, is the average of these three positions:

(43) $$\begin{array}{c} \begin{array}{c} X_{i}^{\mathrm{GWO}}\left(t+1\right)=\frac{X_{1}\left(t+1\right)+X_{2}\left(t+1\right)+X_{3}\left(t+1\right)}{3}. \end{array} \end{array}$$

#### Dimension Learning-Based Hunting (DLH) Strategy

To enhance the search, the DLH strategy introduces a second candidate position. First, a radius $R_{i}\left(t\right)$ is computed using the Euclidean distance between the current wolf’s position and the GWO candidate position:

(44) $$\begin{array}{c} \begin{array}{c} R_{i}\left(t\right)={∥X_{i}\left(t\right)-X_{i}^{\mathrm{GWO}}\left(t+1\right)∥}_{2}. \end{array} \end{array}$$

This radius defines a neighborhood $N_{i}\left(t\right)$ around the current wolf $X_{i}\left(t\right)$:

(45) $$\begin{array}{c} \begin{array}{c} N_{i}\left(t\right)=\left\{X_{j}\left(t\right)\;|\;∥X_{i}\left(t\right)-X_{j}{\left(t\right)∥}_{2}\leq R_{i}\left(t\right),\;X_{j}\left(t\right)\in \mathrm{Population}\right\}. \end{array} \end{array}$$

Then, a new candidate position from the DLH strategy, $X_{i}^{\mathrm{DLH}}\left(t+1\right)$, is generated dimension by dimension:

(46) $$\begin{array}{c} \begin{array}{c} X_{i,d}^{\mathrm{DLH}}\left(t+1\right)=X_{i,d}\left(t\right)+\mathrm{rand}\cdot \left(X_{n,d}\left(t\right)-X_{r,d}\left(t\right)\right), \end{array} \end{array}$$

where $X_{n,d}\left(t\right)$ is the *d*-th component of a neighbor randomly selected from $N_{i}\left(t\right)$, and $X_{r,d}\left(t\right)$ is the *d*-th component of a wolf randomly selected from the entire population.

#### 3.2.2. Objective Function and Problem Formulation

The decision variables that the IGWO algorithm optimizes are the diagonal elements of the Q matrix and the single element of the R matrix. To simplify the optimization, only the parameters for Joint 1 are optimized, and the resulting gains are applied to all joints. The problem has a search dimension of D=4, where each wolf’s position vector $X_{i}$ corresponds to a set of LQR weighting parameters:

(47) $$\begin{array}{c} \begin{array}{c} X_{i}=\left[q_{1},q_{2},q_{3},r_{1}\right] \end{array} \end{array}$$

These parameters construct the weighting matrices as $Q=\mathrm{diag}(q_{1},q_{2},q_{3})$ and $R=[r_{1}]$. The performance of each candidate solution $X_{i}$ is evaluated using a fitness function $f(X_{i})$, which must be minimized. This function is defined as the Integral Absolute Error (IAE) of the joint’s angular position, calculated from a simulation of the closed-loop system using the corresponding Q and R:

(48) $$f\left(X_{i}\right)=\mathrm{IAE}=\int_{0}^{T_{\mathrm{sim}}}\left|\theta_{\mathrm{ref}}\left(t\right)-\theta_{\mathrm{actual}}\left(t;Q,R\right)\right|\;dt,$$

where $\theta_{\mathrm{ref}}$ is the reference angle, $\theta_{\mathrm{actual}}$ is the simulated angle of the joint, and $T_{\mathrm{sim}}$ is the total simulation time.

Finally, the new position for each wolf is determined by comparing the fitness values of the two candidates:

(49) $$\begin{array}{c} \begin{array}{c} X_{i}\left(t+1\right)=\left\{\begin{array}{cc} X_{i}^{\mathrm{GWO}}\left(t+1\right), & \mathrm{if}\;f\left(X_{i}^{\mathrm{GWO}}\left(t+1\right)\right)<f\left(X_{i}^{\mathrm{DLH}}\left(t+1\right)\right),\\ X_{i}^{\mathrm{DLH}}\left(t+1\right), & \mathrm{otherwise}. \end{array}\right. \end{array} \end{array}$$

#### IGWO Pseudo-Code

The overall IGWO procedure for optimizing the LQR weighting matrices is summarized in Algorithm 1.
**Algorithm 1** IGWO for Optimal LQR Weighting Matrices1:**Input:** Population size *N*, dimension *D*, max iterations MaxIter, bounds $l_{j},u_{j}$2:**Output:** Optimal solution vector $X_{\alpha }$ (containing optimal Q and R parameters)3:Initialize the population of wolves ${\left\{X_{i}\right\}}_{i=1}^{N}$ using (35).4:Evaluate the fitness $f(X_{i})$ for each wolf using (48).5:Set iteration counter t←1.6:**while** *t* ≤ MaxIter **do**7:      Identify the top three wolves $X_{\alpha },X_{\beta },X_{\delta }$ based on their fitness values.8:      **for** each wolf $X_{i}\left(t\right)$ in the population **do**9:            Compute the GWO candidate position $X_{i}^{\mathrm{GWO}}\left(t+1\right)$ using (43).10:          Compute the DLH radius $R_{i}\left(t\right)$ using (44).11:          Construct the neighborhood $N_{i}\left(t\right)$ using (45).12:          Generate the DLH candidate position $X_{i}^{\mathrm{DLH}}\left(t+1\right)$ using (46).13:          Select the superior candidate and update the wolf’s position for the next iteration, $X_{i}\left(t+1\right)$, using the selection rule in (49).14:      **end for**15:      Evaluate the fitness of the newly updated positions.16:      Increment iteration counter t←t+1.17:**end while**18:**Return** the position of the alpha wolf, $X_{\alpha }$, as the best solution.

#### 3.2.3. Optimization Implementation and Results

The IGWO algorithm was run for 100 iterations with a population of 50 wolves. The search space for the four decision variables was defined by the following bounds:

- Lower Bounds: l=[0,0,0,0.0001];
- Upper Bounds: u=[500,10,10,2].

After the optimization process, the best solution found corresponded to the following optimal weighting matrices:

$$Q_{\mathrm{optimal}}=\mathrm{diag}\left(498.7,9.98,10\right),\;R_{\mathrm{optimal}}=\left[0.01\right]$$

Using these optimal matrices, the LQR gain matrix K was calculated. This single gain matrix was applied to all three joints:

$$K_{\mathrm{joint}\;1,\;2,\;3}=\left[223.61\;0.629\;0.233\right]$$

## 4. Simulation Results

**Figure 4:**
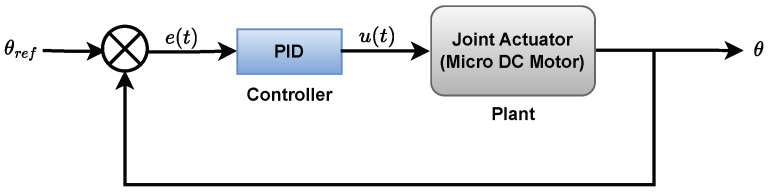
PID schematic diagram.

In this section, the performance of the proposed IGWO-LQR controller is evaluated and compared against two benchmark controllers: a conventionally-tuned Proportional–Integral–Derivative (PID) controller and a PID controller whose gains were optimized using Particle Swarm Optimization (PID-PSO). The basic feedback structure for the benchmark PID controller, where the error between a reference angle ($\theta_{ref}$) and the actual angle (θ) is used to generate a control signal (u(t)), is illustrated in Figure 4. To ensure a fair comparison, separate controllers were implemented for each of the three joints. For the conventional PID benchmark, the gains for Joint 1 and Joint 2 are identical ($K_{p}=0.68,K_{i}=0.001,K_{d}=0.014$), while the gains for Joint 3 are ($K_{p}=0.44,K_{i}=0.01,K_{d}=0.04$). For the PID-PSO benchmark, the PSO algorithm was employed to minimize a cost function defined as the Integral Absolute Error (IAE) of the joint’s angular position. This optimization resulted in gains of ($K_{p}=0.7,K_{i}=0,K_{d}=0.02$) for Joints 1 and 2, and ($K_{p}=0.33,K_{i}=0.27,K_{d}=0.042$) for Joint 3.

The performance of these three control strategies was assessed under four distinct input scenarios to ensure a comprehensive evaluation. In addition to standard step and square-wave inputs that test the response to abrupt changes, sinusoidal and sigmoid inputs were chosen to evaluate performance in scenarios more representative of functional prosthetic use. The sinusoidal input was advantageous for testing the ability to track smooth and periodic motions, which are common in many daily activities. The sigmoid input was used to validate the controller’s capacity for producing smooth, ‘biomimetic’ transitions between joint positions, which is critical for achieving natural and gentle grasping actions.

### 4.1. Response of System Under Step Input

**Figure 5:**
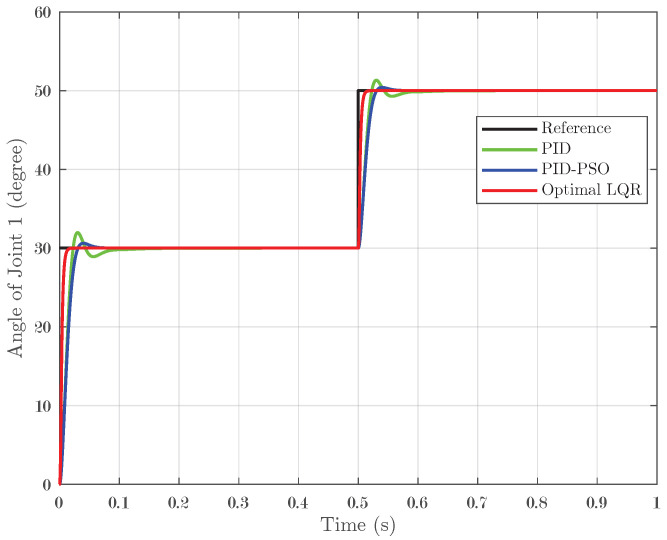
Response of Joint 1 under step input.

**Figure 6:**
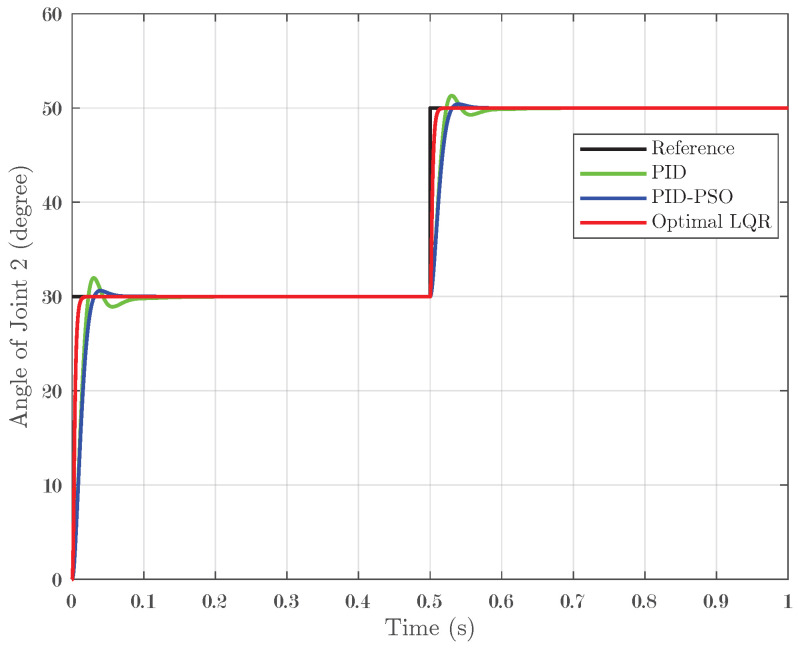
Response of Joint 2 under step input.

**Figure 7:**
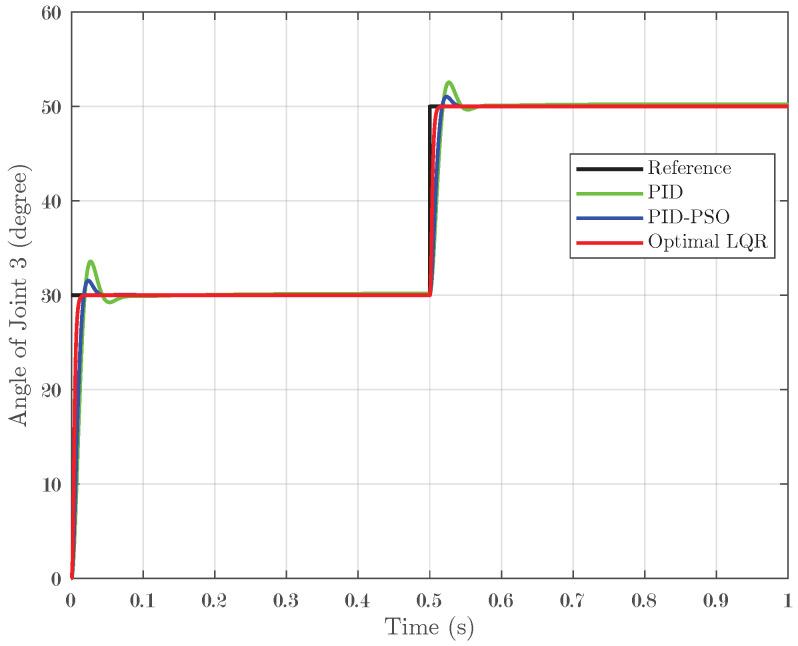
Response of Joint 3 under step input.

In the first simulation scenario, a step input was applied to the system, and the controllers’ performance was evaluated across the three joints. A step test involving a transition from 30° to 50° with zero initial conditions confirmed these trends: Joint 1 (Figure 5) displayed IAEs of 0.01173 for PID, 0.01131 for PID-PSO, and 0.003302 for Optimal LQR; Joint 2 (Figure 6) followed a similar pattern; and Joint 3 (Figure 7) recorded IAEs of 0.01266, 0.007821, and 0.003612 for PID, PID-PSO, and Optimal LQR, respectively. These results clearly demonstrate that the Optimal LQR controller consistently provides faster stabilization, minimal overshoot, and reduced cumulative error compared to the other strategies.

### 4.2. Response of System Under Square-Wave Input

**Figure 8:**
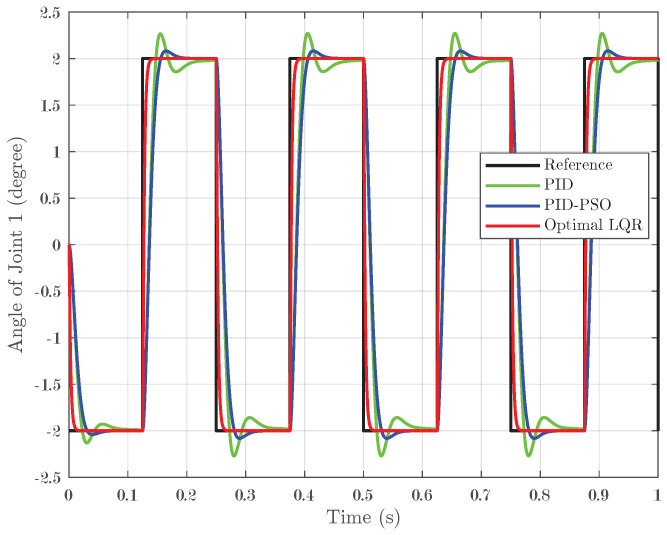
Response of Joint 1 under square-wave input.

**Figure 9:**
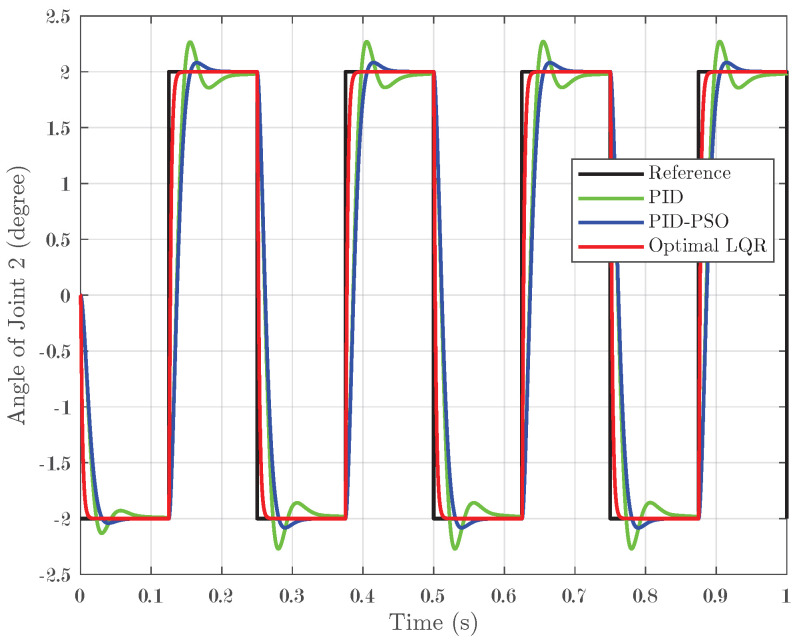
Response of Joint 2 under square-wave input.

**Figure 10:**
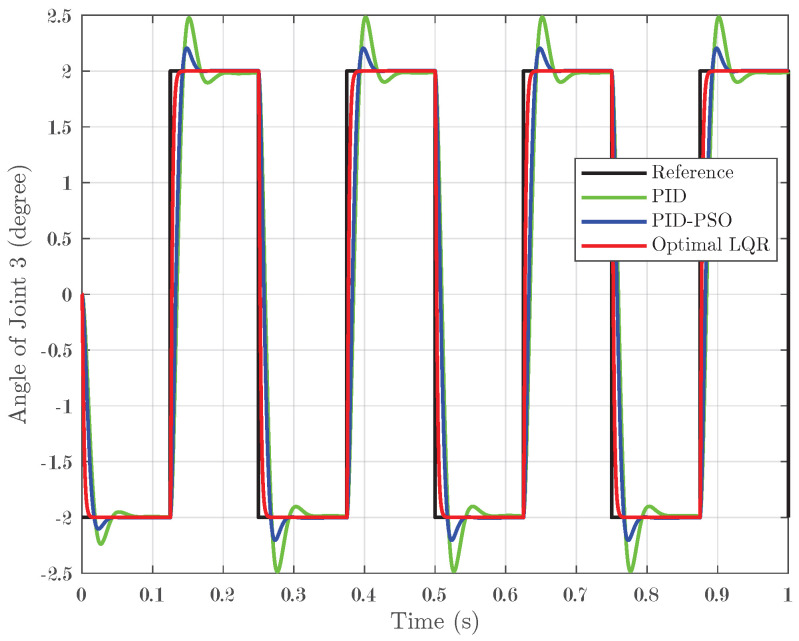
Response of Joint 3 under square-wave input.

The system was further evaluated using square-wave inputs ranging between −2° and 2° with four pulses applied over one second. This test was designed to assess the tracking accuracy of the controllers under rapid, small-angle variations. For Joint 1, as depicted in Figure 8, the Optimal LQR controller demonstrated excellent tracking by closely following the reference square wave and achieving an IAE of 0.001496. In contrast, the conventional PID controller and the PID-PSO controller produced higher IAEs of 0.006921 and 0.006781, respectively, and exhibited more pronounced oscillations during abrupt transitions. Joint 2, as shown in Figure 9, exhibited the same trend, confirming that the Optimal LQR maintains superior accuracy across multiple joints. For Joint 3 (refer to Figure 10), the PID controller recorded an IAE of 0.00628, while the PID-PSO controller achieved an IAE of 0.004588; the Optimal LQR once again outperformed both, with an IAE of 0.001487. These results clearly illustrate that the Optimal LQR controller is more effective in handling rapid, small-amplitude variations, providing smoother and more precise tracking under square-wave inputs.

### 4.3. Response of System Under Sine Input

**Figure 11:**
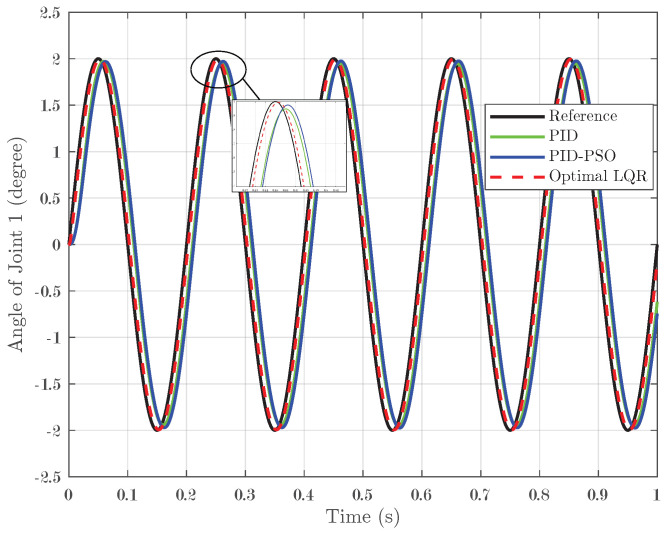
Response of Joint 1 under sine input.

**Figure 12:**
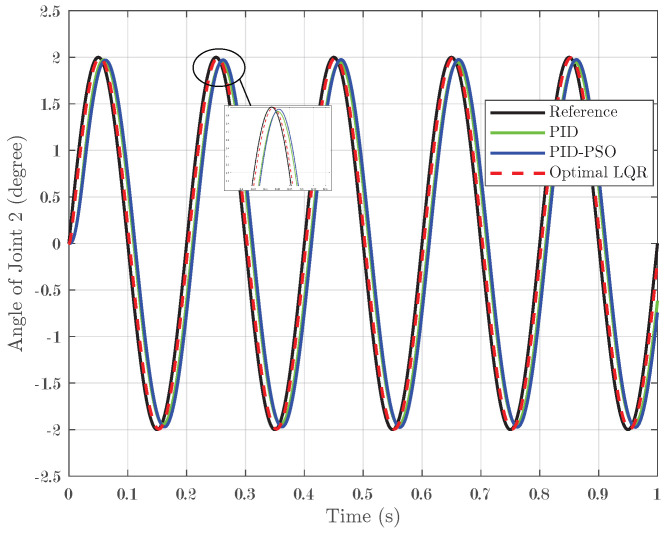
Response of Joint 2 under sine input.

**Figure 13:**
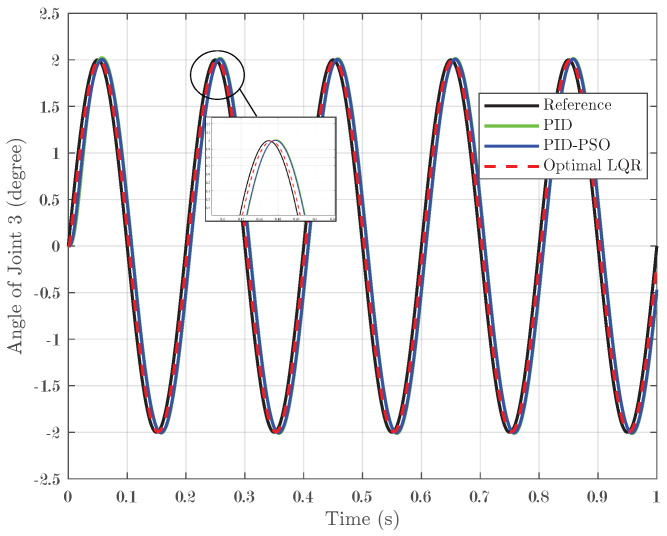
Response of Joint 3 under sine input.

In addition to step and square-wave inputs, the controllers were tested with sine-wave inputs. In this scenario, a sine input with a peak of 2° was applied over one second with five pulses, challenging the controllers to track a continuously oscillating reference signal. For Joint 1, illustrated in Figure 11, the conventional PID controller achieved an IAE of 0.007, while the PID-PSO controller recorded an IAE of 0.0083. The Optimal LQR controller demonstrated remarkable accuracy, with a substantially lower IAE of 0.001975. Joint 2 (shown in Figure 12) exhibited identical performance, reinforcing the consistent efficacy of the Optimal LQR approach across different joints. For Joint 3 (refer to Figure 13), the PID controller produced an IAE of 0.005659, and the PID-PSO controller yielded an IAE of 0.00522; in contrast, the Optimal LQR maintained its high performance with an IAE of only 0.001963. These findings indicate that under sine-wave excitation, where the reference continuously varies, the Optimal LQR controller maintains superior tracking fidelity with minimal error accumulation.

### 4.4. Response of System Under Sigmoid Input

**Figure 14:**
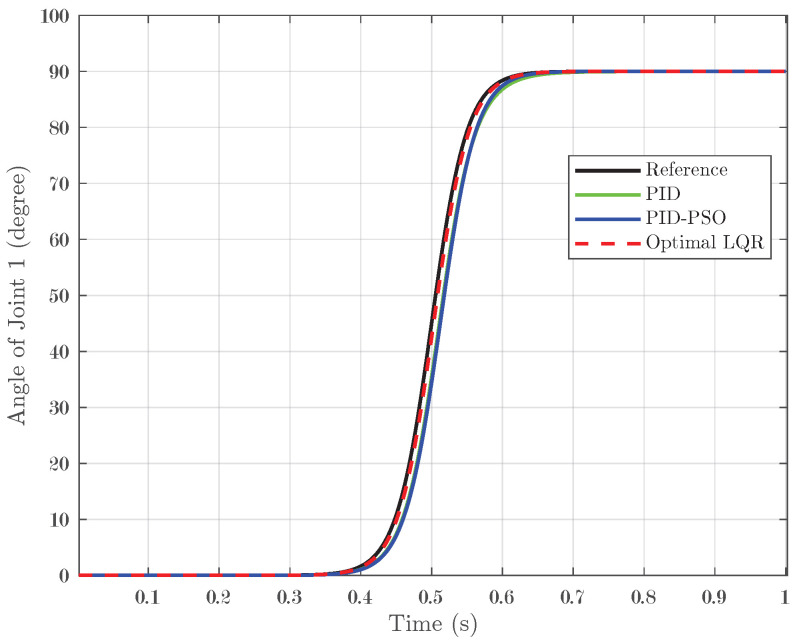
Response of Joint 1 under sigmoid input.

**Figure 15:**
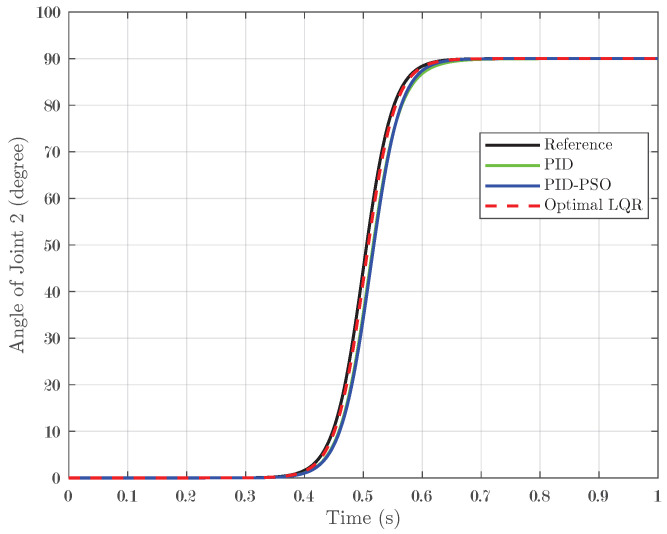
Response of Joint 2 under sigmoid input.

**Figure 16:**
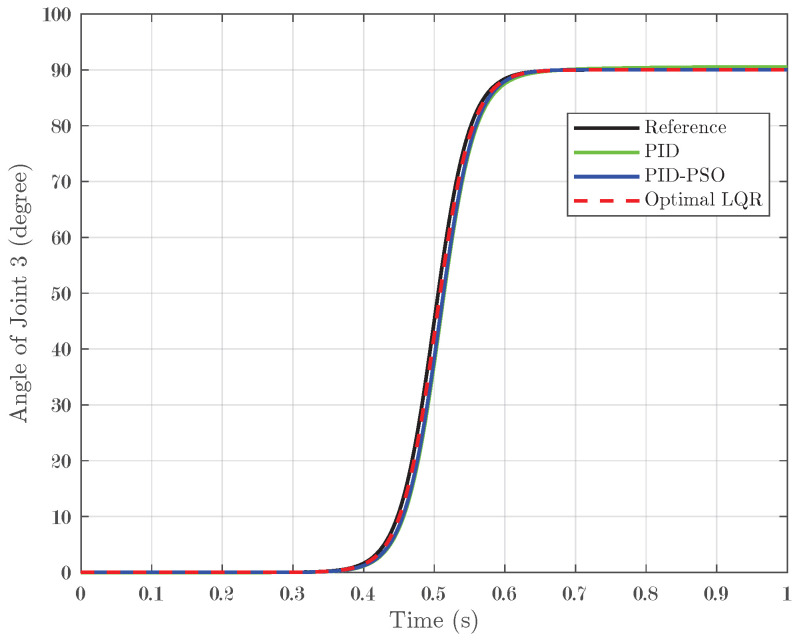
Response of Joint 3 under sigmoid input.

The final test evaluated the system response under a sigmoid input, which transitions smoothly from 0° to 90° and represents a nonlinear, gradual change in the reference signal. For Joint 1 (depicted in Figure 14), the conventional PID controller yielded an IAE of 0.01852, while the PID-PSO controller recorded a similar IAE of 0.01894. In contrast, the Optimal LQR controller significantly outperformed both by achieving an IAE of only 0.004473. Joint 2 (illustrated in Figure 15) showed the same pattern as Joint 1, indicating that the lower error achieved by the Optimal LQR is consistent across these two joints. For Joint 3 (refer to Figure 16), the PID controller registered an IAE of 0.01561, the PID-PSO improved this to 0.01153, and, yet again, the Optimal LQR provided the best performance, with an IAE of 0.004438. This sigmoid test, with its smooth yet nonlinear transition, further underscores the capability of the Optimal LQR controller to adapt to varying input profiles, maintaining minimal error and superior tracking compared to the other control methods.

## 5. Results Discussion

The comprehensive evaluation of the smart finger mechanism under four distinct input conditions—step, square wave, sine, and sigmoid—shown in Table 2 demonstrates the consistent superiority of the Optimal LQR controller, which was optimized via the Improved Grey Wolf technique. Whether confronted with abrupt changes, rapid small-angle transitions, continuous oscillations, or smooth nonlinear transitions, the Optimal LQR consistently achieves faster settling times, eliminates or significantly reduces overshoot, and minimizes cumulative errors (IAE) across all joints. These performance attributes make the Optimal LQR controller particularly well-suited for precision control in complex, multi-joint robotic systems, highlighting its potential for applications where both dynamic performance and steady-state accuracy are critical.

When placed in the context of the broader field, these findings show a marked improvement over conventional control approaches and contribute a valuable design methodology. Much of the foundational work on manipulators for humanoid robots has relied on classical control-system designs [18] that, while functional, often require significant manual tuning and may not achieve the high-speed, low-error performance demonstrated here. Other research has focused on complex, high-level challenges such as inter-finger coordination [20], for which a robust and precise low-level joint controller, like the one developed in this study, is a critical prerequisite. The performance metrics achieved in this work, such as a settling time of 0.018 s, are essential for the viability of advanced systems like the Modular Prosthetic Limb [27], which demand both dexterity and rapid response. The primary advantage of our approach lies in its systematic design framework; by integrating IGWO with the LQR, we automate the process of finding an optimal and robust controller, offering a significant improvement over the manual trial-and-error tuning required in many other control strategies.

## 6. Conclusions

This study successfully integrated an Improved Grey Wolf Optimization (IGWO) algorithm with an LQR controller to optimize the performance of a multi-fingered robotic hand (MFRH). The IGWO-LQR framework addresses the critical challenge of balancing precise trajectory tracking with energy efficiency in prosthetic and assistive systems. Under step-input tests, the proposed controller achieved a settling time of 0.018 s, eliminated overshoot, and significantly reduced the Integral Absolute Error (IAE) for all joints compared to conventional PID and PID-PSO controllers. The controller’s superiority extended to dynamic scenarios, including square-wave and sine inputs, where it maintained rapid response times and minimal cumulative errors, highlighting the efficacy of the metaheuristic optimization approach.

The main contributions and advantages of this research are threefold. First, the proposed IGWO-LQR controller demonstrates unequivocally superior performance in terms of speed, precision, and error minimization compared to standard PID and PID-PSO benchmarks across a variety of motion profiles. Second, this work presents a systematic and automated method for tuning the LQR weighting matrices using IGWO, which overcomes the traditional, often suboptimal, trial-and-error approach to LQR design. Finally, the robustness of the controller, validated on diverse input signals, highlights its potential for reliable operation in real-world prosthetic applications that require complex and varied movements.

Despite these advantages, the study has limitations that pave the way for future work. The primary disadvantage is that the current findings are based entirely on simulation. While the dynamic model is comprehensive, it does not capture all real-world nonlinearities; for example, it does not account for stiction or sensor noise. Furthermore, the current control strategy assumes that the joints are decoupled systems. While effective for the tested scenarios, this simplification does not account for the dynamic coupling effects that exist between the joints of a single finger.

Future work will focus on two key areas: (1) the physical fabrication of the MFRH to test and validate the controller’s performance on a hardware prototype; (2) the development of an enhanced multi-input, multi-output (MIMO) control model that considers the coupling effects between joints, which could lead to more fluid and coordinated multi-joint movements. Successfully addressing these points will further advance the practical viability of this control framework for next-generation assistive devices.

## Figures and Tables

**Table 1:** Finger parameters.

*i*	$\theta_{i}$	$d_{i}$	$\alpha_{i-1}$	$a_{i-1}$
1	$\theta_{1}$	0	0	0
2	$\theta_{2}$	0	$l_{1}\;\left(\mathrm{MCP}\right)$	0
3	$\theta_{3}$	0	$l_{2}\;\left(\mathrm{PIP}\right)$	0
4	0	0	$l_{3}\;\left(\mathrm{DIP}\right)$	0

**Table 2:** Summary of IAE values for different controllers under various input conditions.

Input Type (Joint)	PID IAE	PID-PSO IAE	Optimal LQR IAE
Step (Joint 1)	0.00469	0.00452	0.00124
Square Wave (Joint 1)	0.00692	0.00678	0.00150
Sine (Joint 1)	0.00700	0.00830	0.00198
Sigmoid (Joint 1)	0.01852	0.01894	0.00447
Step (Joint 3)	0.00470	0.00311	0.00133
Square Wave (Joint 3)	0.00628	0.00459	0.00149
Sine (Joint 3)	0.00566	0.00522	0.00196
Sigmoid (Joint 3)	0.01561	0.01153	0.00444

## Data Availability

Data are available on request due to restrictions. The data presented in this study are available on request from the corresponding author. The data are not publicly available.

## Appendix A

**Table A1:** Motor parameters for MFRH joints.

Parameter	Joints 1 and 2 (EC-max 22)	Joint 3 (EC 16)
Nominal Voltage (V)	24 V	24 V
No-load Speed (rpm)	10,500	15,500
No-load Current (mA)	19	11
Stall Torque (mNm)	70	28
Max Continuous Torque (mNm)	24	9
Torque Constant, $K_{t}$ (mNm/A)	8.2	3.2
Terminal Resistance (Ω)	2.85	8.15
Terminal Inductance (mH)	0.072	0.11
Rotor Inertia (g·cm^2^)	6.27	1.33
Weight (g)	66	39
Diameter (mm)	22	16
Length (mm)	45	40

Note: The same motor model is used for Joint 1 and Joint 2.

**Table A2:** Gearhead parameters for MFRH joints.

Parameter	Joint 1 Gearhead	Joint 2 Gearhead	Joint 3 Gearhead
Model	GP 22 C	GP 22 C	GP 16 A
Reduction Ratio (*N*)	64:1	56:1	43:1
Max Continuous Torque (Nm)	1.2	1.2	0.3
Efficiency (η)	70%	70%	70%
Weight (g)	42	42	25
Diameter (mm)	22	22	16
Length (mm)	31.2	31.2	28.1

Note: The same gearhead model is used for Joint 1 and Joint 2, with adjusted reduction ratios.
